# Management of infective endocarditis in a secondary care trust: a service evaluation of treatment outcomes and multidisciplinary involvement

**DOI:** 10.1099/acmi.0.001153.v3

**Published:** 2026-06-03

**Authors:** Lisa Dwyer-Joyce, Mo Chung Kwok, Stephen Hughes, Nupur Goel

**Affiliations:** 1 Chelsea and Westminster NHS Foundation Trust, West Middlesex University Hospital, Twickenham Road, Isleworth, Middlesex, TW7 6AF, UK

**Keywords:** antimicrobial management, infective endocarditis, multidisciplinary team, secondary care, service evaluation

## Abstract

**Background.** Infective endocarditis (IE) is a complex and life-threatening condition, requiring multidisciplinary team (MDT) input for optimal management.

**Objectives.** This study aimed to review the demographics, infection management and clinical outcomes of patients treated for IE at Chelsea and Westminster Hospital NHS Foundation Trust (CWFT), a secondary care provider in London. This study evaluated local IE service provision against national and international guidelines to identify areas for service improvement.

**Methods.** A retrospective cohort study was conducted, identifying adult patients (≥18 years) treated for IE at CWFT between September 2023 and August 2024. Patients who received >10 days of intravenous amoxicillin, flucloxacillin, cefazolin or vancomycin were screened. Patients who received a full course of IE treatment were included. Clinical records were reviewed for demographic, microbiological, imaging, MDT involvement and clinical outcome data.

**Results.** Thirty-one patients met the inclusion criteria. The median age was 77 years (interquartile range 53–85), and 74% were male. *Staphylococcus aureus* (35%) and *Enterococcus faecalis* (29%) were the most common pathogens. Only 12 patients (39%) were discussed at a cardiothoracic MDT, and 2 patients lacked cardiology input. Microbiology consultations occurred in all cases, but only 55% had documented antimicrobial plans. Outpatient therapy was used in 36% of cases. Adverse outcomes of IE treatment included in-hospital mortality in 12.9% (*n*=4) and decline in functional baseline in 29% (*n*=9).

**Conclusions.** Whilst key treatment standards were largely met, gaps in MDT involvement and documentation were identified. Implementation of a dedicated IE MDT may increase adherence to guidelines and improve secondary care service provision.

## Data Summary

The full underlying dataset for this article cannot be shared publicly, as it contains multiple indirect identifiers – including age, gender, treatment location and rare disease characteristics – that could compromise patient privacy. However, all data supporting the findings and conclusions of this study are fully presented within the Results section and accompanying table.

## Introduction

Infective endocarditis (IE) is a complex condition associated with significant morbidity and mortality [1,2]. Due to the complexity of IE treatment, a multidisciplinary team (MDT) management approach is recommended to achieve optimal outcomes [3,5]. National guidance on IE service provision has been outlined by the Joint British Societies (JBS) [6]. Evidence from the Partial Oral Treatment of Endocarditis (POET) trial [7] and international recommendations, including the European Society of Cardiology (ESC) guidelines [8], further inform standards of care.

Chelsea and Westminster Hospital NHS Foundation Trust (CWFT) is a secondary care provider in London, UK, operating across 2 acute sites with ~1,100 inpatient beds and 1 cardiology department. The trust is linked to two external hospitals with a cardiac surgical unit, and patients with suspected or confirmed IE can be referred for discussion at the cardiothoracic MDT. The cardiothoracic MDT comprises specialists in cardiology and cardiothoracic surgery. Referrals are made via a referral system integrated within the trust’s electronic health record.

This study aims to evaluate the IE service provision provided by CWFT against national and international treatment standards.

## Methods

A retrospective cohort study was conducted of adult patients (≥18 years old) who were treated for IE at CWFT between 1 September 2023 and 31 August 2024. Patients who received more than 10 days of intravenous (IV) amoxicillin, flucloxacillin, cefazolin or vancomycin were identified using the trust’s electronic prescribing system. These antibiotics were selected as they form core components of recommended treatment regimens for Gram-positive IE in ESC guidelines [8]. Locally, these agents are used in preference to alternatives such as daptomycin, teicoplanin or benzylpenicillin.

Eligibility was assessed through case-note review. Patients were included if they received a full treatment course of antibiotics for IE or if treatment was continued until death. A full treatment course was defined in accordance with ESC guidelines [8], which recommend 2–6 weeks of antimicrobial therapy for native valve endocarditis and ≥6 weeks for prosthetic valve endocarditis. Patients were excluded if antibiotics were stopped due to a rejected IE diagnosis based on imaging and/or clinical decision.

All included patients were followed up by manual review of clinical records for a minimum of 6 months after completion of IE treatment. Data extracted from clinical records included patient demographics, microbiological findings, imaging results, antibiotic regimens and MDT documentation. The data collection parameters are provided in Material S1, available in the online Supplementary Material.

Follow-up focused on clinical outcomes, including in-hospital mortality, recurrence of infection, treatment-related complications and decline in functional status relative to pre-admission baseline. Decline in functional baseline was considered as an increased level of care required at discharge compared with pre-admission status, for example, discharge to a care facility in a patient previously living independently. Data relating to functional outcomes were obtained from documentation by allied health professionals, such as physiotherapists and occupational therapists, within the clinical record.

Patients transferred to tertiary facilities after 10 days were included; data were collected up to the date of transfer, and additional information regarding post-transfer outcomes was retrieved where available from accessible clinical records.

When analysing the data, continuous variables were summarized as medians with interquartile ranges (IQRs), whilst categorical variables were expressed as frequencies and percentages. All statistical analyses were descriptive in nature.

## Results

### Patient background

A total of 31 patients met the inclusion criteria. The median age was 77 years (IQR 53–85), and 23 patients (74%) were male. IE was clinically suspected on admission in 8 patients (26%), whilst the remaining were initially managed for a non-IE issue. All patients suspected of having IE on admission had three or more sets of blood cultures taken. Overall, 15 patients (48%) had blood cultures taken prior to administration of antibiotics, irrespective of the initial working diagnosis.

Prosthetic heart valves were present in 13 patients (42%), comprising 7 bioprosthetic valves (including transcatheter aortic valve implantation) and 6 mechanical valves. Three patients (10%) had a cardiac implantable electronic device (CIED), 2 patients (6%) had both a prosthetic valve and a CIED, and 13 patients (42%) had no cardiac prosthesis.

Blood cultures grew *Staphylococcus aureus* in 11 patients (35%), of which 10 were methicillin-susceptible and 1 was methicillin-resistant. *Enterococcus faecalis* was found in 9 patients (29%), streptococci in 6 (19%) and coagulase-negative staphylococci in 1 (3%). No causative organism was identified in 4 cases (13%). Table 1 summarizes blood culture results, including the number of positive cultures per patient and the number of cultures obtained prior to initiation of targeted IE therapy.

**Table 1.** Clinical, microbiological and echocardiographic characteristics of patients treated for **IE** Affected valve identified on initial transthoracic echocardiogram (TTE), transoesophageal echocardiogram (TOE) and follow-up TTE imaging, alongside pathogen grown on blood culture for each patient. Additional variables include IE suspicion on admission, timing of blood cultures relative to antibiotic administration, total number of positive cultures and number of blood culture sets obtained prior to treatment.

Patient valve background (native/mechanical/ bioprosthetic/CIED)	Affected valve on initial TTE imaging	Affected valve on TOE imaging	Affected valve on follow-up (within 42 days of treatment initiation) TTE imaging	Pathogen grown on blood culture	Was IE suspected on admission?	Were blood cultures taken prior to administration of antibiotics?	No. of positive blood cultures	No. of blood cultures taken before initiation of IE treatment
Native valve	Tricuspid	Not applicable	Not applicable	*S. aureus*	No	No	4	2
Native valve	Aortic	Not done	Aortic	*S. aureus*	No	No	1	1
Native valve	Aortic	Not done	No IE findings	*S. aureus*	No	Yes	2	2
Native valve	Mitral	Not applicable	Not applicable	*S. aureus*	No	No	3	3
Native valve	No IE findings	Not done	No IE findings	*E. faecalis*	No	Yes	3	3
Native valve	No IE findings	Not done	No IE findings	*E. faecalis*	No	Yes	1	2
Native valve	Mitral	No IE findings	No IE findings	*E. faecalis*	Yes	Yes	5	5
Native valve	Aortic	Not applicable	No IE findings	*E. faecalis*	No	No	1	1
Native valve	No IE findings	Aortic root abscess	Aortic root abscess	*Streptococcus mitis*	No	Yes	1	3
Native valve	Mitral	Not done	Mitral	*Streptococcus gordonii*	Yes	Yes	4	3
Native valve	Aortic, mitral+tricuspid	Not done	Not done	*Streptococcus vestibularis*	No	Yes	2	3
Native valve	No IE findings	Not done	Mitral	No growth	Yes	Yes	0	9
Native valve	Mitral	Not done	No IE findings	No growth	No	No	0	3
Bioprosthetic valve	No IE findings	Not done	No IE findings	*S. aureus*	No	No	2	3
Bioprosthetic valve	No IE findings	Not done	No IE findings	*S. aureus*	No	Yes	2	2
Bioprosthetic valve	Aortic	No IE findings	Not done	*E. faecalis*	Yes	No	2	4
Bioprosthetic valve	No IE findings	Not done	No IE findings	*E. faecalis*	No	Yes	1	1
Bioprosthetic valve	Aortic	Aortic	Aortic	*E. faecalis*	No	No	1	2
Bioprosthetic valve	No IE findings	Not done	Not done	*Streptococcus mutans*	Yes	No	3	3
Bioprosthetic valve	Aortic	Aortic	Aortic	No growth	Yes	No	0	3
Mechanical valve	No IE findings	Not done	No IE findings	*S. aureus*	No	Yes	2	1
Mechanical valve	No IE findings	Not done	No IE findings	*E. faecalis*	No	No	1	1
Mechanical valve	No IE findings	Not done	No IE findings	*E. faecalis*	No	No	1	2
Mechanical valve	No IE findings	No IE findings	No IE findings	*Streptococcus gallolyticus*	Yes	Yes	1	2
Mechanical valve	No IE findings	No IE findings	Not done	Beta-haemolytic strep. Group C/G	No	No	2	2
Mechanical valve	No IE findings	Not done	Aortic root abscess	No growth	No	Yes	0	10
CIED and mechanical valve	Aortic	Not done	No IE findings	*S. aureus*	No	Yes	1	2
CIED and bioprosthetic valve	CIED lead	Not done	No IE findings	*S. aureus*	No	Yes	3	2
CIED	Mitral	Not done	Mitral	*S. aureus*	Yes	No	3	4
CIED	CIED lead	Not applicable	Not applicable	*S. aureus*	No	No	4	3
CIED	Not done	Not done	Not done	*Staphylococcus epidermidis*	No	No	2	1

‘Not applicable’ refers to patients who were unable to receive imaging, e.g. patient transferred to another hospital and patient passed away.

‘Not done’ is when imaging could have been performed but was not, e.g. not requested by the clinical team, patient refusal or deemed not indicated based on cardiology specialist advice.

Transthoracic echocardiography (TTE) was performed in 30 patients (97%), with 16 out of 30 (53%) showing features consistent with IE (e.g. valvular vegetations, aortic root abscess and CIED lead vegetations). Seven patients (23%) had a TOE, with 3 out of 7 (43%) demonstrating signs of IE. Follow-up TTE within 42 days of initiating antimicrobial therapy was performed in 23 patients (74%), of which 8 out of 23 (35%) demonstrated persisting or new evidence of IE. The affected valves identified on imaging for each patient are presented in Table 1.

### Management of IE

Twelve patients (39%) were discussed at a cardiothoracic MDT. Twenty-nine patients received consultations from both cardiology and microbiology specialists; however, the remaining two patients were treated based on microbiology recommendations alone. Specific antimicrobial recommendations were documented in the patient records by the microbiology team in 17 cases (55%).

One patient received incorrect antimicrobial therapy consisting of IV ceftriaxone monotherapy, which did not provide adequate cover for the pathogen identified in their blood culture – *E. faecalis*. The patient was switched from correct therapy to incorrect therapy after a discussion with the microbiology team, although this was documented solely in the ward medical team’s notes. The error was identified by the antimicrobial pharmacist after 2 days and corrected. All the other patients identified in this study were managed with correct antimicrobial therapy as per the ESC guideline IE regimen recommendations [8] or specifically documented microbiology specialist recommendation.

Eight patients (26%) were referred to complete antibiotic treatment via Outpatient Parenteral Antimicrobial Therapy (OPAT), and 3 patients (10%) were switched to oral antibiotics in accordance with the POET trial protocol. Two OPAT patients were subsequently readmitted to complete their antibiotic course as inpatients – one due to persistent fevers and the other due to non-compliance with OPAT appointments.

### Clinical outcomes

Adverse outcomes following treatment included all-cause in-hospital mortality (*n*=4), septic embolic events (*n*=4), all-cause rehospitalization within 2 weeks (*n*=5) and an increase in level of care on discharge compared to pre-admission (*n*=9). Ten patients (32%) experienced no adverse outcomes, whilst outcome data were unavailable for 4 patients who were transferred to external facilities with clinical systems which were not accessible.

Eight patients (26%) were transferred to centres with cardiothoracic surgical services for consideration of surgical management during the initial course of IE treatment. However, information regarding whether surgery was performed, and details of any procedures, were not available for all patients following transfer. No cases of relapse (defined as recurrent bacteraemia with the same pathogen within 6 months) or unplanned emergency cardiac surgery following completion of the initial IE treatment period were recorded across the cohort, including those transferred during treatment, within the available clinical records.

## Discussion

In this retrospective analysis, many patients were male (74%), and over half (58%) had either a prosthetic valve or CIED. This is consistent with established risk factors for IE, with both male sex and cardiac prosthetic devices being associated with increased incidence [9,10].

The identified causative organisms were broadly consistent with global IE epidemiology, with *S. aureus* most common and comparable proportions of streptococcal infection [9,11, 12]. However, *E. faecalis* was the second most frequent organism, reflecting the rising incidence of *E. faecalis* IE reported in other studies, particularly among elderly male patients [13,14], who predominated in our cohort.

### Compliance with recommended standards of care

When benchmarked against recommended treatment standards, several areas of good practice were identified in CWFT’s IE service provision. The ESC guidelines recommend that patients suspected of having IE on admission should have three or more sets of blood cultures taken, and all patients should be reviewed by a microbiologist regarding IE treatment [8]. These standards were consistently met.

However, 52% of patients did not have blood cultures obtained prior to antibiotic administration. This could have potentially led to loss of pathogen detection and increased rates of false-negative results. In addition, significant gaps were identified in MDT input. Two patients did not have a documented cardiology review, and 19 (61%) were not discussed at a cardiothoracic or IE-specific MDT. The JBS guidelines recommend that the IE treating team should include, as a minimum, an infection specialist and a cardiologist, with all cases discussed at an IE MDT [6]. Our findings highlight MDT input as a key area for service improvement.

A potential limitation of the current MDT referral pathway for patients with IE across the trust is that the primary route is via a cardiothoracic surgical MDT, thereby largely restricting referrals to patients being considered for operative intervention. In addition, the cardiothoracic MDT, comprising cardiology and cardiothoracic surgery clinicians, does not include representation from microbiology specialists and therefore lacks dedicated infection expertise in case discussions.

The proposed intervention to address this is the formation of a local MDT involving cardiology, cardiothoracic surgery and microbiology specialists, aiming to discuss all suspected IE cases with clearly documented treatment plans. This approach would enable broader specialist input into treatment decisions and align local practice with JBS recommendations [6]. Similar interventions have been successfully implemented at other institutions [15,16].

### Antimicrobial stewardship and outpatient care

Although all patients received microbiology consultation, just over half had a documented antimicrobial treatment plan from the microbiology team. Furthermore, one patient received incorrect antimicrobial therapy that did not cover the suspected causative organism. Absence of direct documentation by the microbiology team likely contributed to this error. This highlights the need to improve the IE team documentation, with a target for all patients to have a microbiology specialist's documented antimicrobial plan recorded in the clinical notes.

Oral stepdown and OPAT management were utilized in 36% of patients. Whilst these strategies are increasingly used in IE management [7,17], they have risks, and patients need to be carefully selected, as demonstrated by the readmission of a patient following OPAT non-compliance. Utilizing an MDT would allow earlier identification of suitable patients, with improved consensus regarding risk-benefit decisions for outpatient treatment.

### Clinical outcomes

The most frequent adverse IE treatment outcome was a reduction in functional baseline in 29% (*n*=9) of patients, highlighting the substantial morbidity associated with IE in older comorbid patients. Notably, 26% of patients were transferred to a cardiothoracic centre for consideration of surgical management; however, this is likely to be an underestimate due to the inclusion criterion requiring >10 days of antimicrobial therapy, which may have excluded patients transferred earlier in their disease course. In-hospital mortality was 12.9% (*n*=4), which is lower than in other studies [18,19], likely reflecting this selection bias, as discussed in the limitations.

### Limitations

The single-centre retrospective design and small sample size (*n*=31) limit the generalizability of findings and statistical power to detect meaningful differences in outcomes. The inclusion criteria based on receiving >10 days of specific IV antibiotics introduce selection bias. This approach was adopted pragmatically to improve diagnostic specificity; however, it likely excluded patients with severe early complications leading to death or transfer before completing 10 days of treatment. Previous studies have demonstrated that mortality in IE frequently occurs early in the disease course [19,20], highlighting that this methodology may underestimate true mortality rates.

Additionally, patients receiving alternative antibiotic regimens (such as those with Gram-negative IE) were not captured, limiting the comprehensiveness of our cohort and potentially skewing the microbial epidemiology findings.

Data collection was dependent on the completeness and accuracy of clinical documentation, resulting in missing values for several variables. The retrospective nature means that important clinical details, rationale for treatment decisions and informal MDT discussions may not have been adequately captured in clinical records. For example, Duke’s criteria for IE diagnosis were not performed retrospectively due to inconsistent documentation of criteria at the time of diagnosis.

Finally, certain assumptions were required during data extraction. In particular, the timing of antibiotic administration and blood culture collection was inferred from recorded timestamps (e.g. prescribing or sample collection entries), which may not precisely reflect actual administration or sampling times. These factors should be considered when interpreting the findings.

## Conclusion

IE remains a complex condition associated with significant morbidity in secondary care. Multidisciplinary input is required for optimal management. Establishing a dedicated local IE MDT may improve adherence to recommended treatment pathways and facilitate prompt decision-making for this complex condition. Further prospective studies are needed to evaluate the impact of MDT implementation on clinical outcomes in similar settings.

## Supplementary material

10.1099/acmi.0.001153.v3 Supplementary Material 1.

## Abbreviations

CIED: cardiac implantable electronic device
CWFT: Chelsea and Westminster Hospital NHS Foundation Trust
ESC: European Society of Cardiology
IE: infective endocarditis
IQR: interquartile range
JBS: Joint British Societies
MDT: multidisciplinary team
OPAT: Outpatient Parenteral Antimicrobial Therapy
POET: Partial Oral Treatment of Endocarditis
TOE: transoesophageal echocardiogram
TTE: transthoracic echocardiogram

## References

[1] Shah ASV, McAllister DA, Gallacher P, Astengo F, Rodríguez Pérez JA (2020). Incidence, microbiology, and outcomes in patients hospitalized with infective endocarditis. Circulation.

[2] Vincent LL, Otto CM (2018). Infective endocarditis: update on epidemiology, outcomes, and management. Curr Cardiol Rep.

[3] Roy A-S, Hagh-Doust H, Abdul Azim A, Caceres J, Denholm JT (2023). Multidisciplinary teams for the management of infective endocarditis: a systematic review and meta-analysis. Open Forum Infect Dis.

[4] Lau L, Baddour L, Fernández Hidalgo N, Brothers TD, Kong WKF (2025). Infective endocarditis: it takes a team. Eur Heart J.

[5] Elad B, Perl L, Hamdan A, Yahav D, Atamna A (2022). The clinical value of the endocarditis team: insights from before and after guidelines implementation strategy. Infection.

[6] Sandoe JAT, Ahmed F, Arumugam P, Guleri A, Horner C (2023). Expert consensus recommendations for the provision of infective endocarditis services: updated guidance from the Joint British Societies. Heart.

[7] Iversen K, Ihlemann N, Gill SU, Madsen T, Elming H (2019). Partial oral versus intravenous antibiotic treatment of endocarditis. N Engl J Med.

[8] Delgado V, Ajmone Marsan N, de Waha S, Bonaros N, Brida M (2023). 2023 ESC Guidelines for the management of endocarditis. Eur Heart J.

[9] Yew HS, Murdoch DR (2012). Global trends in infective endocarditis epidemiology. Curr Infect Dis Rep.

[10] Tleyjeh IM, Abdel-Latif A, Rahbi H, Scott CG, Bailey KR (2007). A systematic review of population-based studies of infective endocarditis. Chest.

[11] Vogkou CT, Vlachogiannis NI, Palaiodimos L, Kousoulis AA (2016). The causative agents in infective endocarditis: a systematic review comprising 33,214 cases. Eur J Clin Microbiol Infect Dis.

[12] Murdoch DR, Corey GR, Hoen B, Miró JM, Fowler VG (2009). Clinical presentation, etiology, and outcome of infective endocarditis in the 21st century: the International Collaboration on Endocarditis-Prospective Cohort Study. Arch Intern Med.

[13] Escolà-Vergé L, Fernández-Hidalgo N, Larrosa MN, Fernandez-Galera R, Almirante B (2021). Secular trends in the epidemiology and clinical characteristics of *Enterococcus faecalis* infective endocarditis at a referral center (2007-2018). Eur J Clin Microbiol Infect Dis.

[14] Dahl A, Iversen K, Tonder N, Hoest N, Arpi M (2019). Prevalence of infective endocarditis in *Enterococcus faecalis* bacteremia. J Am Coll Cardiol.

[15] Kaura A, Byrne J, Fife A, Deshpande R, Baghai M (2017). Inception of the “endocarditis team” is associated with improved survival in patients with infective endocarditis who are managed medically: findings from a before-and-after study. Open Heart.

[16] El-Dalati S, Cronin D, Riddell J, Shea M, Weinberg RL (2022). The clinical impact of implementation of a multidisciplinary endocarditis team. Ann Thorac Surg.

[17] Durojaiye OC, Morgan R, Chelaghma N, Kritsotakis EI (2021). Clinical predictors of outcome in patients with infective endocarditis receiving outpatient parenteral antibiotic therapy (OPAT). J Infect.

[18] Abegaz TM, Bhagavathula AS, Gebreyohannes EA, Mekonnen AB, Abebe TB (2017). Short- and long-term outcomes in infective endocarditis patients: a systematic review and meta-analysis. BMC Cardiovasc Disord.

[19] Cresti A, Baratta P, De Sensi F, Aloia E, Sposato B (2024). Clinical features and mortality rate of infective endocarditis in intensive care unit: a large-scale study and literature review. Anatol J Cardiol.

[20] Hadji-Turdeghal K, Graversen PL, Møller JE, Bruun NE, Povlsen JA (2025). Patient characteristics, presentation, causal microorganisms, and overall mortality in the NatIonal Danish endocarditis stUdieS (NIDUS) registry. Am Heart J.

