# Supplementary material for: Management of infective endocarditis in a secondary care trust: a service evaluation of treatment outcomes and multidisciplinary involvement
Source: Access Microbiol. 2026 Jun 3;8(6):001153.v3. doi: 10.1099/acmi.0.001153.v3 (PMC13232865; doi:10.1099/acmi.0.001153.v3)
Supplement: Supplementary Material 1. [file acmi-8-01153-s001.pdf]

Supplementary material

Supplementary material 1. Chart review form used to manually extract data from patient clinical records

1. Patient age: _______________
2. Patient sex: _______________
3. Patient valve background: Native valve/ Mechanical valve/ Tissue valve / Cardiac Implantable Electronic Device
4. Was infective endocarditis (IE) suspected on admission? Yes/ No
5. Were blood cultures taken prior to administration of antibiotics? Yes/ No
6. Were three blood cultures taken if IE was suspected on admission? Yes/ No/ Not applicable
7. Pathogen grown on blood culture (if applicable): _______________
8. Number of positive blood cultures: _______________
9. Number of blood cultures taken before targeted IE antibiotic therapy was started: _______________
10. What were the findings of initial transthoracic echocardiogram (TTE) imaging? Signs of IE/ No signs of IE/ Not done
11. Valve shown to be affected on TTE imaging (if applicable): _______________
12. What were the findings of transoespheal echocardiogram (TOE) imaging? Signs of IE/ No signs of IE/ Not done/ Not applicable
13. Valve shown to be affected on TOE imaging (if applicable): _______________
14. What were the findings of follow up echocardiogram imaging (performed within 42 days of initiating antimicrobial therapy)? Signs of IE/ No signs of IE/ Not done/ Not applicable
15. Was the patient stepped down to oral antibiotics? Yes/ No/ Not applicable
16. Was the patient managed through Outpatient Parenteral Antimicrobial Therapy? Yes/ No/ Not applicable
17. Was there a note documented from a microbiology clinician in the patients clinical records regarding infective endocarditis treatment recommendation? Yes/ No
18. Was the patient discussed at a multidisciplinary team meeting? Yes/ No
19. Was the patient referred to a cardiology specialist? Yes/ No
20. Was the patient referred to a microbiology specialist? Yes/ No
21. Was the patient transferred to a centre with a cardiothoracic unit for further management? Yes/ No
22. Was there any of the following negative treatment outcomes (select all that apply):
    I. All-cause in-hospital mortality
    II. All-cause rehospitalisation within 2 weeks
    III. Septic embolism event
    IV. Non-planned emergency cardiac surgery after initial IE treatment course
    V. Relapse of disease (recurrent bacteraemia with the same pathogen within 6 months)
